# Philadelphia-like acute lymphoblastic leukemia: the journey from molecular background to the role of bone marrow transplant—review article

**DOI:** 10.1007/s00277-023-05241-2

**Published:** 2023-05-02

**Authors:** Reham Alghandour, Doaa H. Sakr, Yasmin Shaaban

**Affiliations:** 1 Medical oncology Unit, Oncology Center Mansoura University, Faculty of Medicine, Mansoura University, Mansoura, Egypt; 2 Clinical Hematology Unit, Oncology Center Mansoura University, Faculty of Medicine, Mansoura University, Mansoura, Egypt

**Keywords:** BCR-ABL1, (Ph)-like, ALL, BCR-ABL1-like, JAK, TKI, CRLF2, Allo-HSCT

## Abstract

Philadelphia chromosome-like (Ph-like) ALL is a recent subtype of acute lymphoblastic leukemia. Although it does not express the BCR-ABL fusion gene, it has a behavior like true BCR/ABL1–positive cases. This subtype harbors different molecular alterations most commonly CRLF2 rearrangements. Most cases of Ph-like ALL are associated with high white blood cell count, high minimal residual disease level after induction therapy, and high relapse rate. Efforts should be encouraged for early recognition of Ph-like ALL to enhance therapeutic strategies. Recently, many trials are investigating the possibility of adding the tyrosine kinase inhibitor (TKI) to chemotherapy to improve clinical outcomes. The role and best timing of allogeneic bone marrow transplant in those cases are still unclear. Precision medicine should be implemented in the treatment of such cases. Here in this review, we summarize the available data on Ph-like ALL

## Introduction

Acute lymphoblastic leukemia (ALL) is a common pediatric malignancy, associated with a good prognosis and a high cure rate. In contrast, adult ALL has a more dismal prognosis. That has been attributed to patients’ comorbidities, poor performance status, poor compliance, and higher frequency of poor-risk genomic subgroups [1].

Philadelphia (Ph)-positive chromosome is a genetic translocation between chromosomes 9 and 22 that causes the production of a BCR-ABL1 aberrant fusion gene (BCR, breakpoint cluster region gene; ABL, Abelson proto-oncogene; BCR/ABL. chimeric gene of BCR and ABL) [2] which was reported to be present in 11–30%, and 1–5% of adult and children respectively. This translocation was considered one of the worst prognostic factors before the era of tyrosine kinase inhibitors (TKI) [3]. Rearrangement involving the minor breakpoint in the BCR gene encodes a 190-kDa protein which is more prevalent in both adult and pediatric cases than the major one which encodes a 210-kDa protein [4, 5].

The term “Philadelphia–like” or “BCR/ABL1–like” ALL was defined in 2009 by Boer et al. In their study, they described a subset of ALL with negative (BCR/ABL1, histone-lysine methyltransferase 2 [KMT2A], and transcription factor 3 [TCF3], and pre-B-cell-leukemia transcription factor 1 [PBX1]) but had behavior like “true BCR/ABL1–positive cases” [6]. This was the same observation of Haferlach et al., in 2005 [7]. Later in 2016, the World Health Organization included Ph-like ALL as a novel provisional entity under ALL with known cytogenetic abnormalities and updated in the International Consensus Classification (ICC) of myeloid neoplasms and acute leukemias 2022 [8, 9].

Ph-like ALL may harbor different molecular alterations including (i) rearrangements of CRLF2 (cytokine receptor-like factor 2 receptor), which is the most common; (ii) ABL-class rearrangements; (iii) JAK2, and/or EPOR rearrangements; (iv) other mutation in JAK/STAT signaling; (v) other kinase mutations such as FLT3, NTRK3, PTK2B, and BLNK genes; and (vi) RAS mutations [10, 11]. Notably, all the previously mentioned genes are known to be involved in B cell proliferation, differentiation, and cell cycle regulation. When mutations occur in those genes, constitutive kinase activation would occur through the activation of JAK-STAT, RAS, and ABL1 pathways [12].

**Fig. 1:**
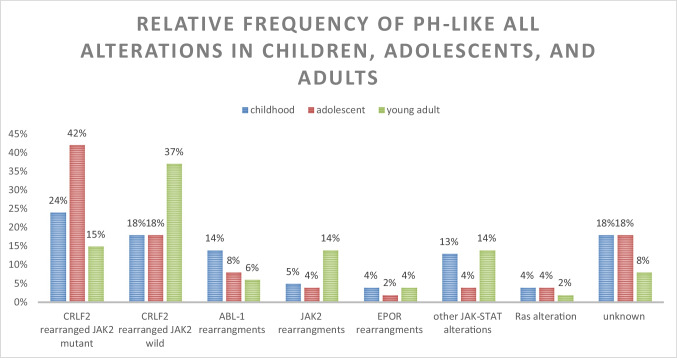
Relative frequency of Ph-like ALL alterations in children, adolescents, and adults, adopted from Tran et al. [14]

The former classification is based upon the similarity of functions of these gene fusions and their potential sensitivity to tyrosine kinase inhibitors (TKI) (e.g., SRC/ABL/PDGFR inhibitors for ABL class fusions and JAK inhibitors for CRLF2, EPOR, and JAK2 rearrangements) [13]. However, JAK inhibitor ruxolitinib could be used in SH2B3 deletions, and TRK (tropomyosin receptor kinase) inhibitors crizotinib and larotrectinib could be used in cases with NTRK fusions [13]. The frequency of the previously mentioned alterations is different according to different age groups; CRLF2 rearrangements are the most common genetic alterations across all age groups. Among children, ABL-class gene rearrangements are more frequent, while among young adults, there is an increase in the frequency of JAK2 rearrangements (Fig. 1) [14].

## Molecular abnormalities in Ph-like ALL

### CRLF2-rearrangements

*CRLF2* rearrangements are the most common rearrangements in Ph-like ALL patients, present in approximately 50% of those patients [15]. The CRLF2 protein is a cytokine receptor that dimerizes with the interleukin-7 receptor (IL7R)-α—located in the sex chromosomes at Xp22.3/Yp11.3. Upon its binding to its ligand, cellular proliferation without differentiation would be provoked through activation of the JAK/STAT and PI3K/AKT/mTOR pathways [16, 17].

Overexpression of CRLF2 is a predictor for poor prognosis in ALL patients [18]. This overexpression in CRLF2 might be due to (i) chromosomal translocation with immunoglobulin heavy-chain locus (IGH)-CRLF2 fusion, (ii) a cryptic interstitial deletion which results in a P2Y receptor family member 8 (P2RY8)-CRLF2 fusion, and rarely (iii) CRLF2 point mutations provoking uncontrolled receptor activation, but sometimes, this may also represent a secondary aberration or a co-occurrence with an established primary lesion, such as the iAMP21, or with high hyper-diploidy [15, 17, 19]. Notably, patients with IGH-CRLF2 have higher CRLF2 expression than those with P2RY8-CRLF2 and, consequently, have a higher risk of relapse rate [20]. CRLF2 deregulation alone is not sufficient to start leukemogenesis; usually, those patients have additional drive mutations like JAK/STAT pathway [21].

Interestingly, it was reported that half the cases of CRLF2-R rearrangements had concomitant JAK2 mutations (most commonly R683G); this entity is named (JAK2 mutant type/CRLF2 rearranged). While (JAK2 wild-type/CRLF2-rearranged) cases show frequent mutations in JAK1, JAK3, FLT3, and the coreceptor IL7R; deletion of the JAK2 negative regulator SH2B3; and translocation IQGAP2-TSLP resulting in CRLF2 ligand overexpression [15, 22]. Notably, the CRLF2-R Ph-like ALL patients with concomitant JAK mutation are mutually exclusive with those who harbor concomitant *IL7R* rearrangements [13]. This group is associated with a worse prognosis [23]. Moreover, IKAROS family zinc finger 1 (IKZF1) is an epigenetic regulator of CRLF2, and the presence of a mutation in IKZF1 leads to overexpression of CRLF2, and this group of patients has a more favorable prognosis [24]. Unfunctionally, IKZF1 alterations are usually accompanied by PAX5 (paired-box 5) alterations, which make the outcome worse [25]. Furthermore, the GATA3 rs3824662 gene risk allele was identified as a susceptibility locus for Ph-like ALL according to the observation of Perez Andreu et al., and others that the ALL patients who harbored GATA3 rs3824662 gene risk allele were associated with *CRLF2* overexpression, JAK mutatio*n*, IKZF1 rearrangement, and those patients had a higher risk of relapse [26, 27]. This observation was confirmed in a cohort of Egyptian children that patients with the GATA3 rs3824662 genotype had poor prognosis with a higher incidence of relapse and short disease-free survival [28].

### ABL-rearrangements

The second common rearrangement in Ph-like ALL is ABL class fusions or translocations which represent nearly 15% of all Ph-like patients with a higher incidence in children than adults [29]. These rearrangements involve the following fusions genes, ABL1, ABL2, CSF1R, PDGFRA, PDGFRB, and FGFR. The presence of any of these translocations is enough to diagnose Ph-like ALL [30]. Notably, these translocations are mutually exclusive with CRLF2 and JAKS/TAT mutations but usually associated with IKZF1 mutations/deletions [17]. The presence of EBF1-PDGFRB (platelet-derived growth factor B) rearrangement has a higher rate of induction failure and measurable minimal residual disease (MRD) [31]. Furthermore, it was reported that acquiring AGGF1-PDGFRB mutation in PDGFRB^C843G^ might be the underlying mechanism of resistance to the ABL TKIs, e.g., imatinib, dasatinib, but could be responsive to multi-target kinase inhibitor like (type II JAK2 inhibitors) CHZ868 [32].

### JAK2 and/or EPOR translocations

Erythropoietin receptor rearrangements (EPOR) and JAK2 rearrangements are nearly present in 7–5% of Ph-like ALL cases; these rearrangements activate JAK-STAT signaling without concomitant CRLF2 alterations [15, 33]. This class comprises JAK2 rearrangements and rearrangements of the EPOR with the immunoglobulin-heavy (IGH) or kappa (IGK) loci that deregulate EPOR expression [34]. This group of patients has the worst prognosis [29]. EPOR rearrangements are 2-fold more common in young adult patients than in children and adolescents [33]. This type of Ph-like ALL usually associated with IKZF1 rearrangement [29, 35].

### Other JAK/STAT pathways and RAS mutation

**Fig. 2:**
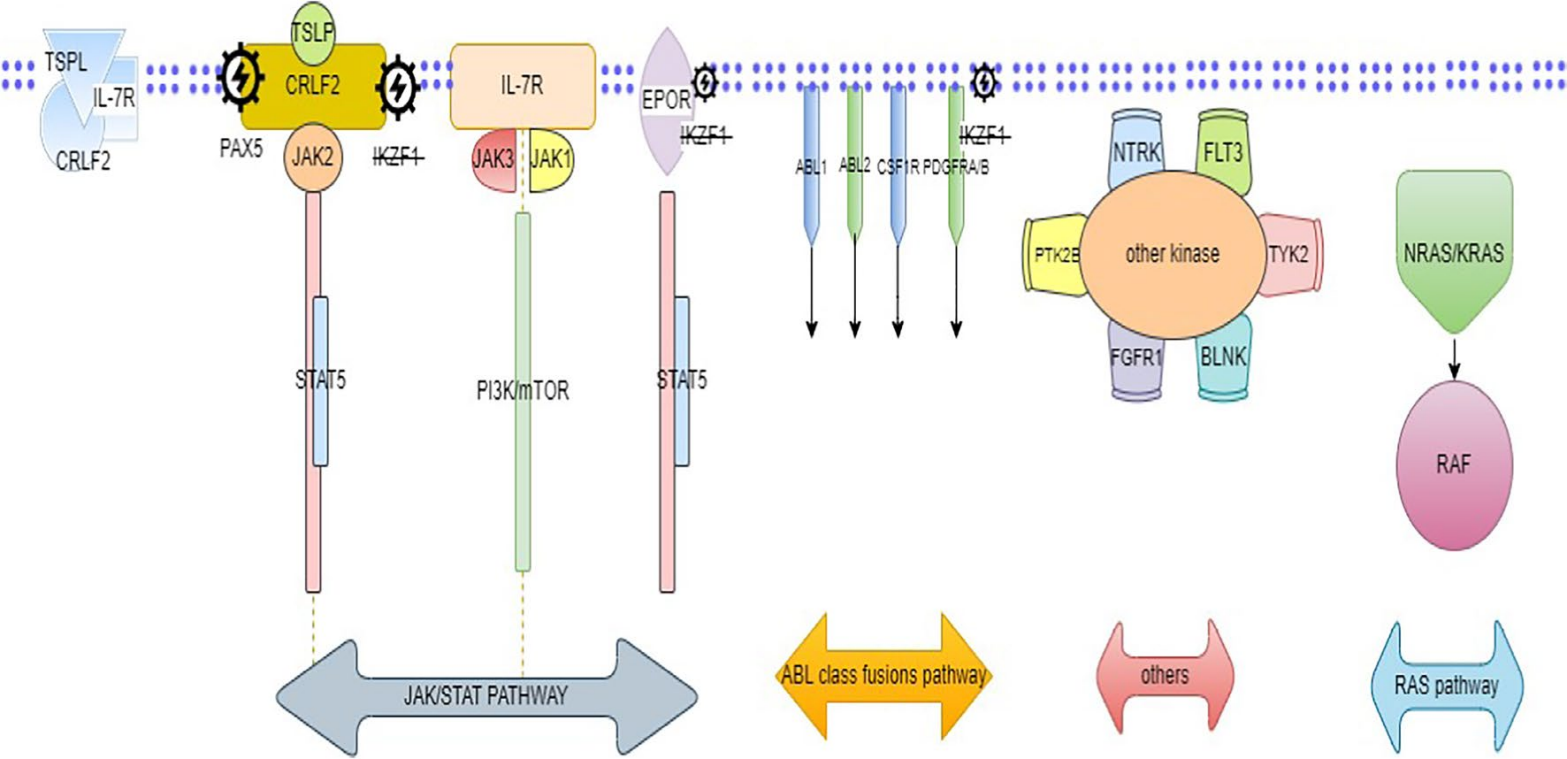
Summary of deregulated molecular pathways regulated in Ph-Like ALL. Caption: CRLF2 with IL-7Rα form heterodimeric receptor complex binds with its ligand TSLP. The signaling pathways observed in Ph-Like ALL including JAK/STAT pathway (involving CRLF2 overexpression with or without JAK2 mutation, IL-7Rα mutation, or EPOR rearrangements) might associate with IKZF1 deletion, ABL fusion signaling pathways, and RAS pathway or involve other kinases

This subgroup represents 15–20% of Ph-like ALL; this group includes alteration of IL7R, SH2B3, JAK1, JAK3, IL2B, FLT3, TYK2, and mutation in RAS pathway (KRAS, NRAS, NF1, PTPN11) [17]. IKZF1 is less common in this subtype of Ph-like ALL than in other subgroups [36]. This subgroup has a better prognosis than other subgroups. RAS pathway mutations may arise in the other subtypes of ALL [17]. Moreover, there were rare rearrangements that had been identified like NTRK3, B-cell linker (BLNK), PTK2B, and TYK2 in Ph-like ALL [25]. These mutations are amenable to being targeted with different types of TKI [37]. A summary of the molecular and genetic pathways is illustrated in (Fig. 2).

## Clinical presentation and outcomes of Ph-like ALL

BCR-ABL-like ALL belongs to the high-risk group of B-ALL in addition to KMT2A (MLL) translocations, low hypodiploidy (30–39 chromosomes), near haploidy (<30 chromosomes), BCR-ABL1, intrachromosomal amplification of chromosome 21 (iAMP21), t(17;19)/TCF3-HLF fusion, and complex karyotype [38, 39]. These groups have dismal prognoses despite modern chemotherapy regimens [22, 40].

The Ph-like ALL represents 10–15%, 20%, and 25–30% of childhood, adults (≥40 years), and adolescents and young adults (AYAs) (age 16–39 years) respectively; the frequency of each kinase subgroup varies with age [41, 42]. Many studies reported that this subtype, Ph-like ALL is usually associated with high white blood cell count (WBC), chemo-resistant, high minimal residual disease (MRD) level after induction therapy, high relapse rate, and short event-free (EFS) and overall survival (OS) [22, 41, 43, 44]. On the contrary, Herold et al. reported no significant differences in baseline patients’ characteristics, including age, sex, WBC count, hemoglobin, or platelet count between the BCR-ABL-like and other subtypes of B-ALL [45]. Also, Saleh LM et al. reported no significant difference in WBC, hemoglobin, platelet count, and sex in patients with higher CRLF2 expression compared to those with low CRLF2 expression [46]. Also, it has been observed that there is a higher incidence of Ph-like ALL in patients with Down syndrome (DS) than in non-Ph-like disease [47, 48].

## Diagnostic approaches to Ph-like ALL

The National Comprehensive Cancer Network (NCCN) guideline version 2.2020 for ALL recommends the evaluation of recurrent genetic and molecular characterization of ALL by using karyotyping of G-banded chromosome analysis, FISH studies for the major recurrent genetic abnormalities, and RT-PCR for BCR-ABL1 (p190 and p210) [38]. In cases of BCR-ABL negative, additional testing is needed for gene fusions and other mutations associated with Ph-like ALL for better risk stratification and management [38]. Array comparative genomic hybridization (array CGH) may be needed if karyotyping failed or aneuploidy is detected [38]. Other methods for genetic characterization include low-density arrays (LDA), next-generation sequencing (NGS)–based assays, and multiplex RT-PCR which are typically used to detect signature or cryptic rearrangements and mutations characteristic of Ph-like ALL [49]. Although the evolution in the techniques of molecular and genetic analysis, there is still debate about the most appropriate approach for the diagnosis and screening of BCR-ABL like ALL. Identification of Ph-like ALL is challenging and usually diagnosed late after completion of induction protocol; major efforts should be done for early recognition of Ph-like ALL by the implementation of CRFL2 immunophenotyping tests and routine application of wide-spectrum, rapid FISH panels, and LDA to enhance therapeutic strategies by involving patients in a clinical trial to add target therapy to the treatment protocol [50].

LDA screening for all ALL patients allows rapid (within 48–72 h) identifying Ph-like ALL patients, those with LDA positive need further genetic testing by FISH or fusion analysis, and/or PCR. TSLPR (CRLF2) flow cytometry is highly cost-effective and can identify the patients within 24 h of specimen acquisition. As CRLF2 (TSLPR) is overexpressed in approximately half of Ph-like ALL cases, the TSLPR flow cytometry is now routinely included in the diagnostic workup of ALL patients. PCR mutation analysis to assess for *JAK2*, *EPOR*, IL-7Rα, and ABL class rearrangements and other rare Ph-like–associated alterations could be further tested [13, 25].

**Fig. 3:**
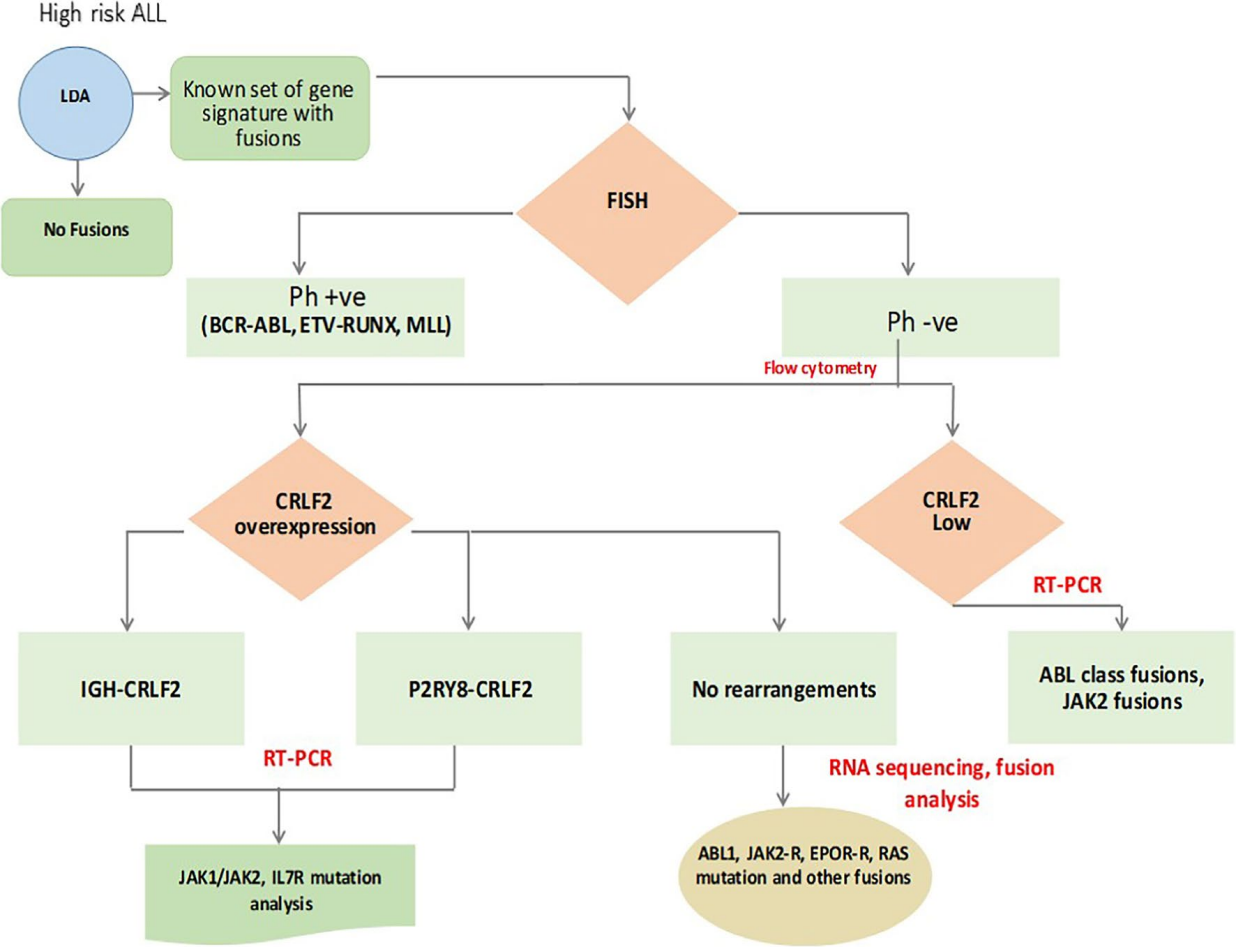
Current Ph-like ALL genetic testing algorithm. Adopted from Yadav et al. LDA screening for all B-ALL patients to rapidly identify those Ph-like ALL from those non-Ph-like ALL; these results are confirmed by FISH testing to rule out those with BCR-ABL1 and ETV6 RUNX1 rearrangements due to similarities in expression signatures; TSLPR flow cytometry immunophenotyping identify patients with overexpression CRLF2-R B-ALL. Confirmatory genetic testing by FISH, fusion analysis, and/or RT-PCR should be performed to characterize the specific CRLF2 alterations, JAK, IL-7Rα, EPOR, and ABL class rearrangements. Abbreviations: ALL, acute lymphoblastic leukemia ; FISH , fluorescence in situ hybridization; LDA, low-density array; Ph-like ALL, Philadelphia-like acute lymphoblastic leukemia; Ph-ve, Philadelphia-negative; Ph+ve, Philadelphia positive; RT-PCR, real-time polymerase chain reaction

Recently, a predictive and statistical model based on Q-RT-PCR has evolved for the identification of Ph-like ALL cases [51, 52]. A stepwise simple diagnostic algorithm adopted from Yadav V et al. summarized the current Ph-like ALL genetic testing, illustrated in (Fig. 3) [53].

## Treatment approaches to Ph-like ALL

The poor outcome of patients with Ph-like ALL and the identification of actionable lesions have opened the way to the treatment of patients with genetic-driven approaches [37]. The NCCN panel recommends that pediatric and AYA patients with Ph-like ALL could be treated in a clinical trial when possible. In the absence of an appropriate clinical trial, the induction therapy consists of multiagent chemotherapy. Patients who have MRD negative after induction will continue risk-stratified therapy, while those with positive MRD after induction may undergo intensified consolidation therapy. If MRD remains persistent, other options include blinatumomab or chimeric antigen receptor (CAR) T-cell therapy tisagenlecleucel. In all cases, an allogeneic hematopoietic stem cell transplant (Allo-HSCT) may be considered part of consolidation or maintenance therapy [38]. A summary of clinical trials conducted on Ph-like ALL patients is illustrated in (Table 1).

**Table 1:** Clinical trials either specific for De novo or R/R Philadelphia chromosome-like acute lymphoblastic leukemia (Ph-like ALL patients) or including patients with Ph-like ALL

NCT number	Group	Schedule	Phase	***N*** patients planned or enrolled	Eligible patients	Age (yrs)	Status
NCT02883049	COG	Dasatinib	3	5956	Newly diagnosed high risk B-ALL, including Ph-like ALL	1–30	Active, not recruiting
NCT02723994	COG	Ruxolitinib with chemotherapy	2	170	children with de- novo High-Risk CRLF2-Rearranged and/or JAK Pathway-Mutant Acute Lymphoblastic Leukemia	1–21	Cohort A: CRLF2-R and JAK+ with EOI MRD ≥0.01%—open to accrual Cohort B: CRLF2-R and JAK– with EOI MRD ≥0.01%—open to accrual Cohort C: JAK2-R, EPOR-R, and SH2B3 mutations and IL7R mutations with EOI MRD ≥0.01%—closed to accrual Cohort D: patients eligible for cohort A, B, or C with EOI MRD
NCT03117751	SJCRH	Ruxolitinib, blinatumomab	2/3	1000	Newly diagnosed patients with B-ALL	1–18	Recruiting
NCT02420717	MDACC	Dasatinib Ruxolitinib	2	92	Relapsed/refractory Ph-like ALL	≥10	Terminated due to low accrual and lack of response
NCT03571321	University of Chicago	Ruxolitinib	1	15	Newly diagnosed Ph-like ALL	18–39.99	Recruiting
NCT03643276	AIEOP/BFM	Bortezomib Blinatumomab	3	5000	Newly diagnosed ALL	≤17	Recruiting
NCT03007147	COG EsPhALL	Imatinib	3	475	Newly diagnosed ALL with ABL class fusion	2–21	Recruiting
NCT03564470	Nanfang Hospital Guangzhou	Chidamide Dasatinib	2	120	Newly diagnosed Ph-like ALL	14–55	Unknown
NCT04501614	COG	Ponatinib	1/2	68	Resistant/refractory Ph + or Ph-like ALL	≥1–21	Active, not recruiting
NCT03834961	COG	Larotrectinib	2	70	Relapsed acute leukemia with TRK fusion	0–≤30	Active, not recruiting
NCT03040030	DFCI	Dasatinib	3	560	De novo B-All with ABL class fusion	≥1–21	Active
NCT03911128	ALLTogether	Imatinib	3	500	Newly diagnosed ALL	≥1–45	Recruiting
NCT02143414	SWOG	Dasatinib	2	57	Newly diagnosed or relapsed Ph-like ALL	≥65	Active, not recruiting
NCT03275493 NCT03614858	Hospital of Soochow University	CART-19/22	1/2	17	Relapsed/refractory ALL	6–65	Active
NCT03181126	Pullarkat et al.	Venetoclax + Navitoclax	1	69	Relapsed/refractory ALL	≥4	Completed

### Induction therapy and the significance of MRD

The induction of remission protocols in ALL usually involves the combination of 4 or 5 of the following drugs: anthracyclines, vincristine, cyclophosphamide, L/PEG-asparaginase, and steroids [17]. Differences between the protocols are in either dosing intensity, schedule, or the addition of 6-mercaptopurine, cytarabine, and rituximab [54, 55]. Based on the success of combining TKI with chemotherapy in cases of Ph + ALL, it raises the question about the validity of using the same approach in the ph like-ALL patients [33], especially since Ph-like ALL is 73 times more resistant to asparaginase and 1.6 times more resistant to daunorubicin and has poor sensitivity to glucocorticoids [6, 56]. In vitro and ex vivo data have reported sensitivity of ABL-class fusions to imatinib or dasatinib, while EPOR, JAK rearrangements, and other activating mutations of the JAK-STAT pathway can be effectively inhibited by JAK inhibitors such as ruxolitinib [57]. Other rare kinase alterations in Ph-like ALL can also be targeted by crizotinib, FAK inhibitors for NTRK3, PTK2B fusions, and TYK2 inhibitors for TYK2 fusions [29, 33].

However, among 148 adult B-ALL patients (including Ph-like, Ph-positive, and other B-ALL) treated with hyper-CVAD or augmented BFM (Berlin-Frankfurt-Munich) protocols with no specific intensification or modification for their high-risk ALL, in spite that CR rate was similar in the three studied groups, but MRD negativity was less achieved in the Ph-like ALL patients and translated to significantly worse overall survival (OS) and event-free survival compared to other B-ALL with a 5-year survival of 23% versus 59% for other B-ALL [23]. A new comprehensive study from the UK has suggested that the cutoff level for clinically relevant MRD is different for various genetic subtypes of ALL. Thus, it is reasonable to consider all Ph-like ALL patients as high risk, regardless of their MRD status [58]. Some data from 344 pediatric patients suggest that therapy intensification for Ph-like MRD^+^ patients can lead to MRD eradication and improve outcomes. However, confirmation from additional large studies is needed to adopt the best induction chemotherapy protocol to be used in those patients [10].

Although it is not specifically tested in the Ph-like ALL, the addition of rituximab if leukemic cells are CD20^+^ and the use of L- or PEG-asparaginase, known to be active in high-risk ALL, may be recommended [17]. At the same time, the risk of asparaginase-related complications at this age needs to be considered [59].

Blinatumomab, a bispecific antibody targeting CD19 and CD3, has not yet been tested as an agent for Ph-like ALL treatment intensification. However, it has been proven to be effective in MRD eradication and is currently being incorporated in clinical trials as part of front-line treatment for other high-risk ALL patients [60]. Furthermore, Meyer et al. observed that ALL with CRLF2 overexpression demonstrated suboptimal response to glucocorticoid in vitro, and this sensitivity might be augmented when MEK inhibitor trametinib and Akt inhibitor MK2206 combined with glucocorticoid but not the JAK inhibitor ruxolitinib [56]. Achieving MRD negativity is less likely in patients with Ph-like ALL, but the impact of persistent MRD and the intensification of therapy (including the use of Allo-HSCT) to deal with persistent MRD is not clear [44].

### Role of target therapy

#### Targeting activated JAK-STAT signaling

The JAK1/2 inhibitor ruxolitinib is currently being studied in clinical trials for Ph-like ALL patients who have *CRLF2* rearrangements or other JAK pathway alterations. Early-phase clinical trials have reported the safety and tolerability of combining JAK inhibitors with chemotherapy [37].

Koppikar et al. reported resistance to type 1 JAK inhibitors in CRLF2 rearranged B-ALL cells due to paradoxical hyperphosphorylation of JAK2 which produces a state of persistent JAK2 signaling. However, in contrast to JAK I inhibitors, type JAK II inhibitors stabilize JAK2 in the inactive conformation and overcome the JAK2 hyperphosphorylation observed with type I inhibitors, suggesting that type II JAK2 inhibition may be a more effective strategy to target CRLF2-rearranged B-ALLs. But high ruxolitinib doses of at least 50 mg twice daily might be needed to achieve clinical benefit [61, 62].

Meanwhile, the clinical benefit of the addition of ruxolitinib to chemotherapy in ALL is still questionable [33]; however, in the COG ADVL1011 phase 1 trial, the safety and tolerability of ruxolitinib monotherapy were demonstrated in children with multiply-relapsed/refractory cancers, and a recommended phase 2 dose of 50 mg/m^2^ twice daily for 28 days/cycle was identified [63]. Maude et al. reported poor response to single-agent ruxolitinib in preclinical sitting [64], but in further studies conducted by Bӧhm et al., ruxolitinib enhanced the in vivo efficacy of an induction regimen consisting of vincristine, dexamethasone, and L-asparaginase in CRLF2-rearranged Ph-like ALL xenografts [57].

Phase II studies exploring the role of incorporating ruxolitinib in induction regimens for Ph-like ALL are still ongoing (NCT03117751, NCT03571321, NCT02723994, and NCT02420717) [65–67]. CHZ868 is a type 2 JAK inhibitor molecule, investigated in mouse models of CRLF2-rearranged B-ALL; it could induce apoptosis and improve survival in those models [68].

In addition, recent studies showed strong effects of combinatorial treatment with JAK1/JAK2 and PI3K/mTOR inhibitors. In patient-derived Ph-like ALL murine xenograft models, cotreatment with PI3K/mTOR inhibitor gedatolisib and ruxolitinib and gedatolisib and dasatinib had superior efficacy than any of the agents alone [37]. Similarly, the combination of next-generation inhibitors such as type II JAK inhibitor (NVP-BBT594) and second-generation mTOR inhibitor (AZD2014) induced robust anti-leukemic effects in Ph-like ALL cell lines and PDX models harboring CRLF2 rearrangements ±JAK mutations [69].

The cell surface expression of CRLF2/TSLPR, which forms a functional heterodimeric complex with IL7R, is also being exploited for immunotherapeutic targeting with the development of anti-CRLF2/TSLPR antibodies and TSLPR-directed CAR-T cell therapy [70].

#### Targeting ABL-class fusions

For the Ph-like patients presenting with ABL rearrangements, data are suggesting that inhibition by BCR/ABL-specific TKI may be beneficial [17, 32]. It had been reported a sustained response of Ph-like ALL patients with ABL-class fusions to imatinib or dasatinib, particularly those harboring rearrangements of PDGFRB, which is associated with induction failure and a dismal outcome [71].

In the COG AALL1131 (NCT02883049) trial, dasatinib was tested in patients with confirmed ABL-class alteration, daily dasatinib was added to augmented BFM-based chemotherapy at the start of consolidation and continued until the end of maintenance therapy, and the results are still awaited [49]. Moreover, interim data analysis of phase 1/2 study conducted at MDACC reported the safety and efficacy of adding dasatinib to hyper-CVAD chemotherapy in adolescents and adults with relapsed/refractory Ph-like ALL and ABL class fusions (NCT02420717 ) without identified dose-limiting toxicity [13, 72].

Although there is no high-level evidence regarding the value of TKI in patients who have ABL rearrangement, given the established safety and efficacy of these drugs in Ph-positive ALL patients, off-label TKI use could be considered in the treatment of Ph-like ALL than the use of JAK inhibitors in patients harbor JAK rearrangements [17].

#### Targeting NTKR3 fusions

One uncommon but recurrent alteration identified in approximately 1% of Ph-like ALL is ETV6-NTRK3. TRK fusions have been identified in breast carcinoma, infantile sarcoma, acute myeloid leukemia, and more recently pediatric glioma [73]. In vitro and in vivo treatment with the TRK inhibitor larotrectinib demonstrated specific and durable reduction of leukemic burden below detectable levels [74]. Larotrectinib received Food and Drug Administration (FDA) approval in 2018 for the treatment of adult and pediatric patients with solid tumors that have an NTRK gene fusion [75]. Nardi et al. reported a substantial response by using larotrectinib in a refractory Ph-like ALL patients harboring NRAS mutation and ETV6-NTRK3 rearrangement after the failure of CAR T cell therapy [76]. Thus, screening for ETV6- NTRK3 in newly diagnosed ALL and testing the clinical efficacy of TRK inhibition should be considered [77, 78].

#### Targeting Ras/MAPK pathway alterations

Up to 6% of patients with Ph-like ALL have mutations in the Ras/MAPK signaling pathway as their main abnormality [29]. For Ph-like ALL with Ras mutations, Ras inhibition failed, as it is impossible to inhibit Ras directly, but targeting the Ras pathway downstream effectors such as MEK inhibitors represents a new therapeutic strategy [33].

#### Targeting SMAC (apoptotic regulator)

Birinapant, a small molecule mimetic of the apoptotic regulator (SMAC), acts as a mitochondria-derived activator of caspase (SMAC) mimetics targeting inhibitor of apoptosis proteins which activates cell death pathways [79]. Birinapant enhanced the antileukemic activity both as a single agent or in combination with an induction-type regimen of vincristine, dexamethasone, and L-asparaginase against Ph-like ALL xenografts; it was found that chemo-resistant Ph-like leukemic cells were acutely sensitive to Birinapant [80].

## Role of hematopoietic stem cell transplantation based on MRD status

The benefit of allogeneic hematopoietic stem cell transplantation (Allo-HSCT) is outbalanced by the high therapy-related mortality associated with the procedure. So, the appropriate selection of patients with high relapse risk and the lowest risk of transplant-related complications is important. As such, because Ph-like patients are associated with a high risk of relapse, Allo-HSCT might be of value as a consolidation modality for these patients, especially in patients who expected to have a low risk of transplant-related complications. However, there is insufficient data on the outcomes of Allo-HSCT for Ph-like ALL, and it remains debatable whether all adult Ph-like ALL patients should receive an allogeneic HSCT in the first CR, irrespective of other indications So far, no recommendations on the use Allo- HSCT have been established in Ph-like ALL patients in the CR1 [44, 81].

Robert et al. reported no difference in the outcome of Ph-like patients treated with intensified consolidation (including Allo-HSCT) and non–Ph-like pediatric or adult patients [10, 41]. Cho H et.al. reported that Ph-like ALL patients who received Allo-HSCT after CR1 were not inferior in relapse rate, DFS, and OS compared to standard risk ALL but had better relapse rate, DFS, and OS compared to other poor risk cytogenetics ALL [82]. However, in a more recent Chinese retrospective study, the overall response rate of Allo-HSCT after 1 month of TKI was 100% compared to 62% and 73% after 1 month of TKI treatment combined with chemotherapy, and CAR-T cell therapy respectively. Moreover, Allo-HSCT is associated with better DFS and OS compared to CAR-T cell therapy in univariate analysis [83].

MRD positivity after the induction therapy is associated with worse survival and a high risk of relapse in patients with Ph-like ALL as well as other types of ALL [84]. Allogeneic SCT is strongly recommended in this setting [85]. Furthermore, no difference in overall survival in ALL patients who achieved MRD negativity at the end of the induction protocol whatever its type (i.e., in Ph-positive, Ph-like, Ph-negative ALL); also, there were no statistically significant differences in outcomes between the three previous groups after Allo-HCT in CR1 [84, 86] that endorse the significance of MRD negativity exceed the cytogenetic risk group [86, 87].

However, since the results of Allo-HSCT are superior in patients who are MRD-negative before the transplant, intensification of therapy aiming to eradicate the residual disease is logical even before allogeneic HSCT [88]. Remarkably, patients with Ph-like ALL are more likely to still be MRD-positive at the end of the induction protocol [84, 86]. FDA specifically approved the use of blinatumomab for high-risk B-ALL patients who achieve remission but remain MRD positive. In a prospective trial, MRD was eliminated in 78% of patients following blinatumomab. Poor outcome was reported in ALL patients above 15 years old, who were MRD positive after the initial therapy and receive no blinatumomab before allogeneic HSCT, which was confirmed in a large European retrospective analysis [89]. Despite that the previous trials did not screen for Ph-like ALL, it is assumed that blinatumomab could have similar efficacy in Ph-like ALL [90].

Several reports demonstrated complete eradication or significant reduction of MRD at the time of Allo HSCT resulting from pre-transplant intensification with ruxolitinib [67]. However, the addition of targeted therapy, such as JAK or BCR/ABL inhibitors, should not substitute MRD eradication with blinatumomab or intensive chemotherapy before transplantation. Currently, no data are available to support maintenance with JAK2 inhibitors following allogeneic SCT in Ph-like ALL patients [67]. For patients with Ph-like ALL who carry an ABL rearrangement, the addition of post-transplant TKI maintenance is still questionable because of the rarity of this condition. However, safety data of post-transplant TKI maintenance can be extrapolated from the Ph-positive ALL, and its use could be encouraged [91]. This is in agreement with what was reported by Niswander et al., as they reported a substantial response to the addition of ruxolitinib or ponatinib to post-induction chemotherapy; one patient achieved MRD-negative remission by adding ponatinib to blinatumomab and then underwent Allo-HSCT followed by 2 years of maintenance ponatinib posttransplant [66].

Persistence of MRD even after allogeneic SCT or failure to eradicate it before transplantation is a poor prognostic marker and a sign of impending relapse. In these circumstances, patients should be aggressively treated with intensive therapy, and CAR T-cell therapy should be considered. A clinical trial with focal adhesion kinase (FAK) inhibitors could be an appropriate option for patients with IKZF1 mutations or deletions [92].

However, due to the lack of evidence supporting routine assignment to allogeneic SCT, a recent expert review and recommendations from the European Working Group for Adult Acute Lymphoblastic Leukemia (EWALL) and the Acute Leukemia Working Party of the European Society for Blood and Marrow Transplantation (EBMT) recommended the use of allogeneic SCT during first complete remission only in MRD-positive pediatric and adult patients with Ph-like ALL [93].

Notably, a higher rate of relapse is observed in adult Ph-like ALL patients even in MRD-negative cases than in pediatric patients with identical MRD kinetics of eradication. Therefore, Allo-HSCT should be considered in adult Ph-like ALL patients especially those with IKZF1 alteration or other high-risk alterations even if they achieve molecular remission [94].

**Fig. 4:**
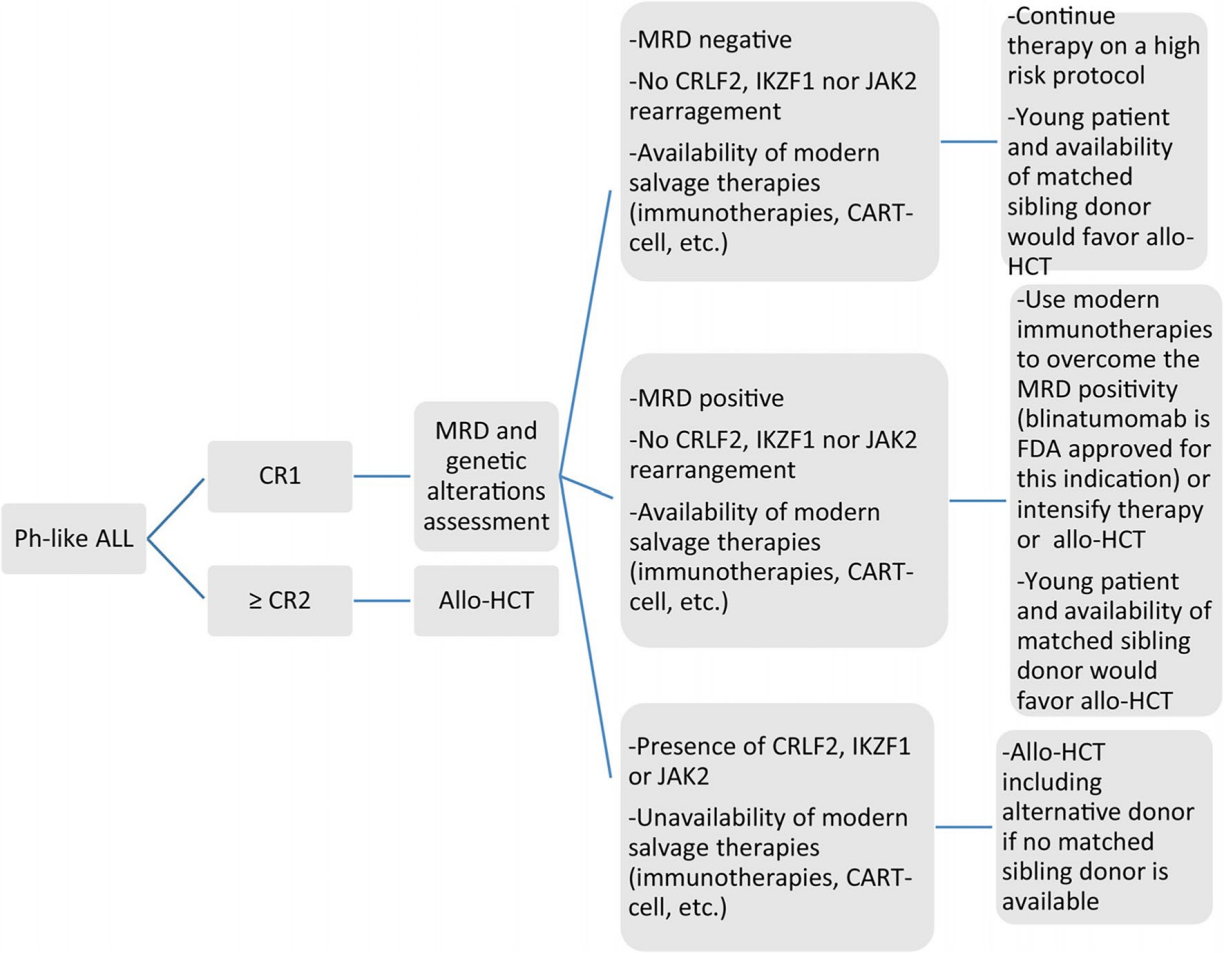
Proposed algorithm by El Fakih et al. to address the role of allogenic bone marrow transplant in the Ph-like ALL patient. Adopted with his permission from El Fakih et al. [44]

EL Fakih et al. suggested an algorithm on when to consider Allo-HSCT for Ph-like ALL patients, illustrated in (Fig. 4) [44].

## Management of relapsed patients

In CD19^+^ ALL, blinatumomab is an acceptable option for both adult and pediatric patients. Inotuzumab ozogamicin (InO) is a humanized anti-CD22 monoclonal antibody also an accepted option for adult patients. CAR T-cell therapy is a powerful strategy to be used in such high-risk patients followed by Allo-HSCT [95]. Combining blinatumomab with inotuzumab is also a good option but should be evaluated against the risk of developing the veno-occlusive disease during subsequent Allo-HSCT [96]. A phase I study by Pullarkat et al. used venetoclax (selective BCL2 inhibitor), with low-dose navitoclax, a BCL-XL/BCL2 inhibitor, in combination with chemotherapy in relapsed/refractory ALL patients, and reported promising outcomes within all genomic subtypes, although in Ph-like and Ph-positive ALL, responses were fewer. The complete remission rate was 60%, including responses in patients who had previously undergone Allo-HSCT or received immunotherapy [97]. A recently published trial conducted by Dai et al. investigated CAR T-cell therapy followed by allogeneic stem cell transplantation, and they reported that the response rate was 94.1%; MRD negative CR was achieved in 11 out of 17 patients, with estimated rates of 3-year overall survival (65.9%±16.5%); the results were comparable in among the included Ph-like, Ph+, and other B-ALL groups [98].

## Management of old age patients

Although Ph-like ALL was reported in 24% of ALL patients over the age of 65 in the USA, no prospective studies have included these patients. On the other hand, Herold et al. showed that the incidence of Ph-like ALL decreased significantly with more advanced age [99].

Unfortunately, for most patients with advanced age, prolonged intensive chemotherapy followed by Allo-HSCT is not feasible [100].

In a study conducted at MD Anderson Cancer Center on a group of 52 patients with a median age of 68 years, inotuzumab ozogamicin was used instead of a significant portion of chemotherapy, creating a less toxic first-line regimen. The 2-year progression-free survival (PFS) was 59% [101].

## Conclusion

Ph-like ALL is one of the high-risk ALL, characterized by a high relapse rate, short event-free, and overall survival. Accurate and rapid identification of these patients is critical to determine the treatment plan. The inclusion of those patients in clinical trials should be encouraged to better understand the benefit of the addition of TKI to the induction treatment protocol. The role of allogeneic bone marrow transplant in Ph-like All in CR1 is still unclear; however, this decision should be tailored according to MRD status.

## Data Availability

This is a comprehensive review, extensive electronic search on google scholar, Pubmed, Medline, and Egyptian Knowledge Bank done using the keywords and all the available data in the literature.

## Abbreviations

*ALL*: acute lymphoblastic leukemia
*Ph-like*: Philadelphia chromosome-like
*TKI*: tyrosine kinase inhibitor
*BCR*: breakpoint cluster region gene
*ABL*: Abelson proto-oncogene
*Allo-HSCT*: allogenic hematopoietic stem cell transplant
*KMT2A*: histone-lysine methyltransferase 2
*TCF3*: transcription factor 3
*TSLP*: thymic stromal lymphopoietin receptor
*PB**X1*: pre-B-cell-leukemia transcription factor 1
*CRLF2*: cytokine receptor-like factor 2 receptor
*IKZF**1*: IKAROS family zinc finger 1
*PDGFRB*: platelet-derived growth factor B
*MRD*: minimal residual disease
*EPOR*: erythropoietin receptor rearrangements
*JAK2*: Janus kinase 2
*WBC*: white blood cell count
*EFS*: event-free survival
*OS*: overall survival
*DS*: Down syndrome
*NCCN*: National Comprehensive Cancer Network
*LDA*: low-density arrays
*NGS*: next-generation sequencing
*FISH*: fluorescence in situ hybridization
*RT-PCR*: real-time polymerase chain reaction
*CAR-T*: chimeric antigen receptor (CAR) T-cell therapy
